# Association between Obesity, Race or Ethnicity, and Luminal Subtypes of Breast Cancer

**DOI:** 10.3390/biomedicines10112931

**Published:** 2022-11-15

**Authors:** Kalhara R. Menikdiwela, Chanaka Kahathuduwa, Michelle L. Bolner, Rakhshanda Layeequr Rahman, Naima Moustaid-Moussa

**Affiliations:** 1 Department of Nutritional Sciences, Texas Tech University, Lubbock, TX 79409, USA; 2 Obesity Research Institute, Texas Tech University, Lubbock, TX 79409, USA; 3 Department of Psychiatry, School of Medicine, Texas Tech Health Sciences Center, Lubbock, TX 79430, USA; 4 Agendia Inc., Irvine, CA 92618, USA; 5 Breast Cancer Center of Excellence, Texas Tech Health Sciences Center, Lubbock, TX 79430, USA

**Keywords:** breast cancer, obesity, race, Luminal A, Luminal B

## Abstract

Luminal breast cancers are the most common genomic subtype of breast cancers where Luminal A cancers have a better prognosis than Luminal B. Exposure to sex steroids and inflammatory status due to obesity are key contributors of Luminal tumor development. In this study, 1928 patients with Luminal A breast cancer and 1610 patients with Luminal B breast cancer were compared based on body mass index (BMI), age, race, menopausal status, and expressed receptors (i.e., estrogen (ER), progesterone (PR), and human epidermal growth factor receptor 2 (HER2)). Patients with Luminal B tumors had a significantly higher mean BMI (Δ = 0.69 kgm^−2^ [0.17, 1.21], *p* = 0.010) versus Luminal A. Interestingly, the risks of Luminal B tumors were higher among Black/African American patients versus White and Hispanic patients (*p* < 0.001 and *p* = 0.001, respectively). When controlled for each other, Black/African American race (*p* < 0.001) and increased BMI (*p* = 0.008) were associated with increased risks of Luminal B carcinoma, while postmenopausal status was associated with a decreased risk (*p* = 0.028). Increased BMI partially mediated the strong association between Black/African American race and the risk of Luminal B carcinoma. Thus, Black/African American race along with obesity seem to be associated with an increased risk of more aggressive Luminal B breast carcinomas.

## 1. Introduction

The precise etiology of most breast cancers is uncertain due to the complexity and diversity of breast tumor subtypes [1,2,3]. For example, meta-analyses have shown increased breast cancer risk among postmenopausal women [4,5,6] and numerous studies have demonstrated a link between endogenous hormone exposure (e.g., estrogen and progesterone) and hormone receptor-positive breast cancers [7,8,9]. In addition, despite the observations of racial disparities in breast cancer, these associations are poorly understood [10]. Several studies suggest that both race or ethnicity and endocrine-related pathways involved in carcinogenesis may be associated with the progression of certain breast cancer molecular subtypes [11,12]. Molecular classification of breast cancers based on genomic profiling can differentiate Luminal cancers (mostly impacted by endocrine pathways) from HER-2-enriched and Basal-like cancers in terms of therapeutic response and prognosis [13]. Therefore, it is imperative for future studies exploring the etiological pathways and therapeutic targets to focus specifically on genomic profiles for precise data.

Luminal cancers are typically estrogen receptor-positive cancers that make up almost 70% of all breast cancers [14]. Typically, Luminal A breast cancer tumors are ER and PR receptor-positive but negative for HER2 [15]; while Luminal B breast cancers are ER and HER2 receptor-positive but negative for PR [16]. Two main biological processes are involved: (i) proliferation-related pathways and (ii) luminal-regulated pathways distinguish Luminal-like tumors into Luminal A and B subtypes with different clinical outcomes [17]. Breast cancer associated with obesity (BMI ≥ 30) is mostly linked to ER and PR receptor-positive cancers rather ER-negative breast tumors in postmenopausal women, allowing treatments using anti-hormone therapies [6]. Obesity is a complex disease associated with various physiological and molecular changes that result in metabolic dysregulation, such as insulin resistance, cell stress (oxidative and endoplasmic reticulum stress), and excess inflammatory cytokine secretion [18,19,20]. These metabolic alterations and physiological changes generate an environment that enriches cancer development [21]. Whether these metabolic changes impact proliferation pathways and luminal-regulated pathways differently can only be understood if the association with obesity is studied in the context of the molecular profiling of cancer. Therefore, it is important to understand the relationship between obesity and its associated inflammatory response as well as race or ethnicity and in the context of the genomic profiles of breast cancers. For the present study, we examined the associations between genomically profiled breast cancers and obesity. This study provides evidence to explore potential pathways impacted by pathophysiological changes associated with obesity and their role in racial disparities that could potentially enhance the personalized strategic approach to the prevention and treatment of breast cancer.

## 2. Materials and Methods

### 2.1. Study Population

The FLEX registry (NCT03053193) enrolled 4530 patients from 14 December 2018 to 5 September 2020 across 90 institutions in the United States. The protocol was approved by institutional review boards at all participating sites. This study was conducted in accordance with the ethical standards established in the Declaration of Helsinki. All patients consented to study participation, clinical data collection, and publication. Patients who were diagnosed with histologically proven early-stage breast cancer (stage I–III) and received MammaPrint genomic testing, and BluePrint genomic testing as a standard of care, were eligible for inclusion. The age range of patients was 18–90 years. Patients consented to the acquisition of clinical data and clinically annotated full-transcriptome tumor analysis. Treatment decisions were made at the discretion of primary treating physicians.

### 2.2. Molecular and Clinical Subtyping

MammaPrint and BluePrint are based on microarray gene expression analysis [22,23] performed at the Agendia Laboratory (Irvine, CA, USA). MammaPrint categorizes tumors as low risk (MammaPrint index > 0.000) or high risk (MammaPrint index ≤ 0.000) of distant recurrence. BluePrint classified tumors into Luminal-type, HER2-type, or Basal-type. MammaPrint further stratified Luminal-type into Luminal A-type (low risk) or Luminal B-type (high risk).

### 2.3. Statistical Methods

All statistical analyses were performed using R statistical software (version 4.1.3). Patients with Luminal type A and Luminal type B breast cancers were compared based on the following characteristics: BMI, age, menopausal status, and race, using *t*-tests with Welch–Satterthwaite continuity correction or chi-squared tests with Yate’s continuity correction. Univariate logistic regression models were constructed to predict patients with Luminal B (vs. Luminal A) breast cancer using each of the above predictor variables. In addition, informed by the initial comparison of prevalence of the two types of breast cancer in among different ethnicities, the two groups were also specifically compared for the proportion of Black/African American individuals. All considered variables were included in a multivariate logistic regression model to predict patients with Luminal type B breast cancer. The model with the lowest Akaike Information Criterion was identified in a stepwise forward selection approach using the caret package in R (version 6.0.90). Informed by the best-fitting model and the literature, it was hypothesized that the association between Black/African American race and Luminal B cancer is mediated by BMI. The validity of this model was examined in a mediation analysis performed via Sobel’s method [24,25] using the mediation package in R (version 4.5.0). Confidence intervals for direct and indirect (i.e., via BMI) effects of the association between Black/African American race and Luminal B breast cancer were computed via a non-parametric bootstrap approach (1000 iterations) using the mediation package in R. One major assumption of causal mediation analysis is sequential ignorability (i.e., the lack of an unmeasured confounder that is associated with both the outcome and the concerned mediator). Unfortunately, as with most studies that employ causal mediation analyses, the assumption of sequential ignorability cannot be tested using the archival data available in our current study. We performed sensitivity analyses to ensure that our results were robust to any violation of this assumption and thus to provide evidence regarding the generalizability of the model [26]. In the sensitivity analyses, an unmeasured theoretical confounder was computed, which correlates with the mediator and outcome. The strength of the correlation between the unmeasured confounder and the mediator and outcome was designated by the sensitivity parameter (*ρ*). Average causal mediation effects (ACMEs) were computed to sensitivity parameters ranging from −1 to +1. An additional exploratory mediation analysis was performed utilizing the same methodology to examine whether mediation of the association between Black/African American race and Luminal B breast carcinoma holds true after controlling for the menopausal status of the patients. In addition, simple univariate and subsequent multiple exploratory logistic regression analyses were performed to examine the associations between estrogen, progesterone, and HER2-neu receptor positivity and Luminal B (vs. Luminal A) breast cancers.

## 3. Results

### 3.1. Characteristics of Patients with Luminal A vs. Luminal B Breast Cancer

The FLEX registry database used for the analyses included 1928 patients with Luminal A-type breast cancer and 1610 patients with Luminal B-type breast cancer at the time of data lock. Exploratory comparisons of patients with Luminal A vs. Luminal B tumors revealed a significantly lower mean age (Δ = 1.25 years [95% CI 0.42, 2.08], *p* = 0.003) and a significantly higher mean BMI (Δ = 0.69 kgm^−2^ [95% CI 0.17, 1.21], *p* = 0.010) in those with Luminal B tumors compared with Luminal A (Table 1). Considering that the accepted threshold of a ≥ 5% change in BMI improves the health-related quality of life, a mean BMI difference of 2.25% is not clinically meaningful. The proportion of postmenopausal women was significantly lower among patients with Luminal B tumors when compared to Luminal A (Table 1; *p* = 0.026). Confirming these findings, univariate regression models indicated that increasing BMI increases the odds of Luminal B breast cancer (Table 2, Model 1, *p* = 0.010), while increasing age and postmenopausal status decreased the odds of Luminal B breast cancer (Table 2, Models 2–3, *p* = 0.003 and *p* = 0.023, respectively).

### 3.2. Effect of Race on Luminal B Breast Cancer

The proportion of Luminal A vs. Luminal B carcinomas by race or ethnicity was significantly different (Table 1; *p* < 0.001). A logistic regression model of patients with Luminal B (vs. Luminal A) breast cancer using race, including Black/African American race as the reference category revealed that Latin American and White patients have lower odds of having Luminal B carcinoma compared to Black/African American patients (Table 2, model 4). When we compared patients of Black/African American race to all other races in a univariate logistic regression analysis, there was a significant increase in the odds of Black/African American women having Luminal B cancer compared to other races (Table 2, model 5; *p* < 0.001).

### 3.3. Predicting the Risk of Luminal B vs. Luminal A Cancer Using Demographic Variables

A multivariate logistic regression model that included BMI, menopausal status, and Black/African American race as predictors was identified as the model of best fit in predicting the likelihood of having Luminal B cancer based on the Akaike Information Criterion (Table 2, Model 6). All regression coefficients in this model were significant and, according to this multivariate model, when controlled for each of the considered variables, an increased BMI (*p* = 0.008) and Black/African American race (*p* < 0.001) were associated with increased odds of having Luminal B carcinoma, while postmenopausal status (*p* = 0.028) was associated with decreased odds of having Luminal B carcinoma.

### 3.4. Analyses Examining Whether BMI Mediates the Association between Black/African American Race and Luminal B Breast Cancer

**Figure 1:**
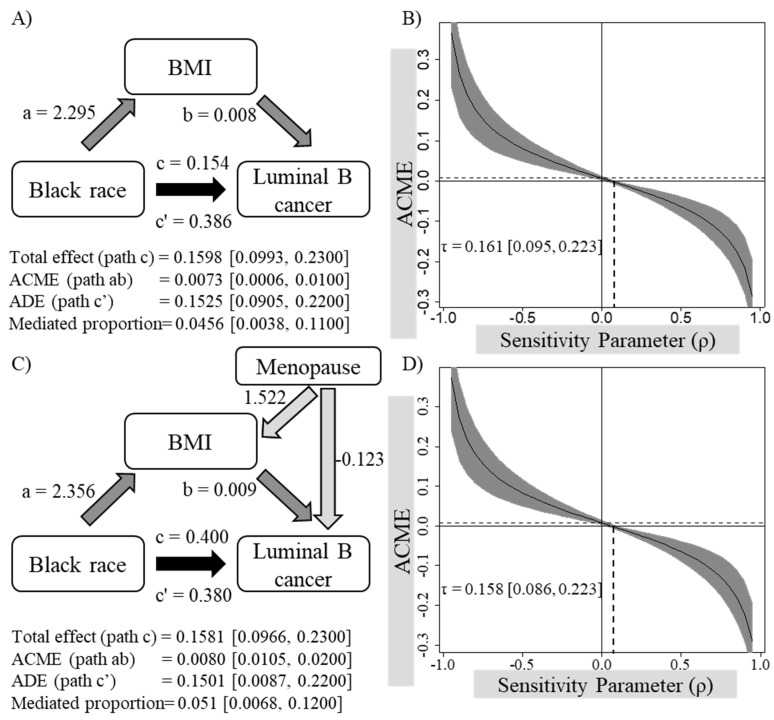
Model diagrams (**A**,**C**) and sensitivity plots (**B**,**D**) of the mediation analyses examining whether the association between Black/African American race and Luminal B breast carcinoma is mediated by BMI before (**A**,**B**) and after (**C**,**D**) controlling for the menopausal status. ACME, average causal mediation effect; ADE, average direct effect; a, regression coefficient between Black/African American race and BMI; b, regression coefficient between BMI and Luminal B cancer when controlled for Black/African American race; c, regression coefficient between Black/African American race and Luminal B cancer; c’, regression coefficient between Black/African American race and Luminal B cancer when controlled for BMI; τ, sensitivity parameter required to nullify ACME. Confidence intervals have been obtained using a non-parametric bootstrap approach (1000 simulations). Light colored arrows arising from the covariate Menopause represent the regression coefficients between Menopause and each outcome variable (i.e., BMI and Luminal B cancer) in each model.

A mediation analysis was performed to examine whether the observed strong association between the Black/African American and Luminal B breast carcinoma is mediated by BMI (Table 3; Figure 1A). Black/African American race was a significant predictor of both Luminal B breast carcinoma (Table 3, Model 1, β = 0.154, *p* < 0.001) and higher BMI (Table 3, Model 2, β = 2.295, *p* < 0.001). When controlled for BMI, the association between Black/African American and Luminal B carcinoma remained significant (Table 3, Model 3, β = 0.386, *p* < 0.001). Using a non-parametric bootstrap analysis, the average causal mediation effect (ACME), as well as the average direct effect (ADE), were significant (Figure 1, 0.0073 [95% CI 0.0006, 0.0100] and 0.1525 [95% CI 0.0905, 0.2200], respectively). However, it should be noted that the average direct effect was significantly greater than the indirect effect. The association between the sensitivity parameter and the ACME of the model is shown in Figure 1B. An unmeasured confounder with a sensitivity parameter of ≥0.161 [95% CI 0.095, 0.223] was required to result in a negative ACME, indicating the robustness of the model. These findings suggest that the association between Black/African American and the odds of Luminal B breast carcinoma in relation to Luminal A carcinoma seem to be partially mediated by relatively higher BMI among Black/African American women.

An exploratory mediation analysis between Black/African Americans and Luminal B breast carcinoma mediated by BMI and a covariate of menopausal status confirmed our findings (Table 3; Figure 1C). Confidence intervals generated using non-parametric bootstrap analyses confirmed that the ACME and ADE in this model were also significant (0.0080 [95% CI 0.0010, 0.2000] and 0.1501 [95% CI 0.0875, 0.2200], respectively), suggesting that the association between Black/African American and Luminal B breast carcinoma appears to be partially mediated by BMI even after controlling for the menopausal status. Sensitivity analyses confirmed that the model holds true in the absence of an additional confounder that is correlated with the mediator and outcome at *ρ* ≥ 0.158 [95% CI 0.086, 0.223] (Figure 1D).

## 4. Discussion

In the current study, we examined associations between obesity and race with breast cancer risk across BluePrint Luminal subtypes. Here, we revealed that the prevalence of the more aggressive Luminal B carcinoma subtype was strongly associated with race, where the odds of having Luminal B cancer were significantly higher among Black/African American women compared to other races or ethnicities including White and Latin Americans.

Breast cancer is a heterogeneous disease where its prognosis and etiology are determined by many factors, including a correlation with race or ethnicity [27,28,29]. Our results noted a significant difference between ethnicity and the proportion of Luminal A vs. Luminal B carcinoma. Luminal B tumors have more aggressive clinical and biological features with a lower expression of estrogen and progesterone receptors, making them less sensitive or effective for endocrine therapies compared to Luminal A tumors [30]. Incidence rates of Luminal A and Luminal B tumors and the association with age has been evaluated but a few studies have evaluated the rates of tumor Luminal subtypes (Luminal A or Luminal B) among racial groups [28,31]. We demonstrate that Latin and White American patients possess a lower probability of having Luminal B carcinoma compared to Black/African American patients.

In line with our findings, Troester MA et al. reported that Black/African American women have significantly statistically higher odds of having Luminal B (OR = 1.45, 95% CI = 1.02 to 2.06) tumors compared to white women [28]. However, the odds ratio was attenuated after adjusting for other clinical covariates, such as tumor size, nodal status, stage, and grade [28]. Several studies have demonstrated that Black/African Americans have a higher incidence of more aggressive breast cancer subtypes. For instance, significantly higher rates of triple-negative breast cancer prevalence were observed in Black/African American women compared White women [27,29,32]. Additionally, certain ethnic groups with breast carcinoma may have lower survival rates [33,34]. According to recent findings, among women with triple-negative breast cancer, Black/African Americans had a higher mortality rate (28% increased risk of death) compared non-Hispanic White women with triple-negative breast cancer [33]. On the other hand, a population-based cross-sectional study carried out recently revealed that Luminal B breast cancer incidence rates increased in all age groups for non-Hispanic White and Hispanic women, with no statistical differences in the Black/African American group, which contrasts with our findings. Whereas the causes of these observed survival difference in Black/African American women could be due to differences in access to screening and treatments and socioeconomic factors, the prevalence of high genomic profiles related to biological differences, including genetic variations across different ethnicities, is less clear. Our findings suggest that race (specifically Black/African Americans) could be a vital factor for the prognosis of more aggressive breast carcinomas. Hence, it would be worth considering race as a parameter along with age (the current recommendations in breast cancer screening is based on the age) when initiating breast cancer screening as certain ethnic groups may have higher risk for breast cancer even at early ages of life [35].

Similar to race, our results also indicated a potential link between BMI and Luminal B carcinoma. Elevated BMI (obese or overweight) increases the risk of numerous cancers, including breast cancer [36,37]. Metabolic alterations due to the overaccumulation of fat in the adipose tissue located in the breast may produce a favorable environment for the development or progression of cancer [21]. Adipokines and inflammatory cytokines secreted by adipose tissue play a vital role in regulating pathways, such as Janus kinase/signal transducer and activator of transcription (Stat) signaling, phosphoinositide 3-kinases/ AKT Serine/Threonine Kinase (PI3K/Akt) and mitogen-activated protein kinase/ Mitogen-Activated Protein Kinase 1 (MAPK/ERK), which promote cancer cell survival, and proliferation [38,39,40,41]. A meta-analysis revealed that obese women with breast cancer have poorer survival rates compared to women with breast cancer who are not obese. Additionally, obesity significantly increased the risk of more aggressive tumors, including triple-negative and Luminal B carcinomas, particularly in premenopausal women [42,43]. We observed that when controlled other variables including menopausal state, Black/African American race and increased BMI was associated with Luminal B carcinoma. Similarly, a consistent relationship between BMI and Luminal B tumors was also demonstrated in a cross-sectional study [44]. Nevertheless, this linear relationship between BMI and the probability of being diagnosed with Luminal B breast cancer demonstrated by Brouckaert O et al. was only seen in postmenopausal women [44]. However, an inverse association was observed among premenopausal women for obesity and breast cancer incidence. A meta-analysis in over 2.5 million women and 7930 premenopausal breast incidences revealed that the breast cancer risk is reduced by roughly 8% for every 5 kg/m^2^ BMI increase in premenopausal women, indicating an interesting association between breast cancer and a patient’s age and BMI, [45]. It could be plausible that there are other underlying factors (such as hormonal status, the level of adipokine-like Adiponectin, and functionality of adipose tissue in early obesity vs. obesity at later stages) that influence the breast tumor progression. Therefore, additional clinical research focusing on the role of adipose tissue in pre- and postmenopausal women in different age groups and BMI categories is warranted to properly identify the relationship between obesity and breast carcinoma.

Although we identified several interesting associations across race, BMI, and Luminal B carcinoma prevalence, our findings should be interpreted in light of some limitations. In the current study, the proportion of Black/African Americans with Luminal A (5.8%) and Luminal B (10.2%) tumors are considerably lower compared to the portion of White Americans in these breast cancer subtypes (Luminal A 72.0% and Luminal B 67.7). This could be due to the smaller Black/African American population size in the area or a smaller number of participants among women from lower socioeconomic and educational levels. This also highlights disparities in the under-representation of Black/African Americans in clinical trials and human subjects’ studies. These factors may directly affect the cancer incidence rate and could produce an underestimation of the prevalence of Luminal B breast cancer in Black/African Americans. However, a similar proportion of Latin Americans (2.6% of Luminal A cases and 2.1% of Luminal B) showed lower odds of having Luminal B, partially eliminating the effect of a lower population size in certain racial groups on the incidence rate of the Luminal B cancer subtype. Yet, the small sample size is one of the limitations as the Black/African American portion considered in the current study is not an exact representation of the entire Black/African American population in the United States. Therefore, further research with an increased sample size (especially Black/African Americans) is needed to confirm the finding that the odds of having Luminal B cancer was significantly higher among Black/African American women.

## 5. Conclusions

Despite these limitations, the findings of this study clearly demonstrate that race and obesity could be a decisive factor for more aggressive breast carcinomas (Luminal B), even though certain biological differences can be observed across different subtypes of breast cancers. At present, The US Preventive Services Task Force (USPSTF) breast cancer screening recommendations do not reflect or consider race as a factor in cancer screening [35]. Projections of the size and composition of the US population predicts major drifts and changes in certain racial or ethnic minority populations by 2050 [46]. Therefore, it would be worth considering race along with age and revising breast cancer screening guidelines in the future. More importantly, as we develop new therapies to treat Luminal B cancers, the pathophysiological pathways associated with obesity and drug metabolism differences among racial profiles would be important to consider in future studies.

## Figures and Tables

**Table 1:** Patient characteristics based on breast cancer type.

	Luminal A		Luminal B		Δ [95% CI]/Proportion ‡	t/χ^2^ ‡	df	*p*-Value
	Mean/N †	SD/% †	Mean/N †	SD/% †				
BMI	29.86	7.10	30.55	7.52	−0.69 [−1.21, −0.17]	−2.583	2890.6	0.010
Age	61.43	11.12	60.18	12.51	1.25 [0.42, 2.08]	2.950	2922.2	0.003
Menopause	1340	0.82	1057	0.79	0.56	4.982	1	0.026
Race						25.254	7	<0.001
Black/African American	112	0.06	165	0.10	0.40			
Asian	38	0.02	37	0.02	0.51			
Latin American	93	0.05	73	0.05	0.56			
Native American	4	0.00	3	0.00	0.57			
Pacific Islander	3	0.00	3	0.00	0.50			
Other	35	0.02	30	0.02	0.54			
Unknown	253	0.13	210	0.13	0.55			
White	1390	0.72	1089	0.68	0.56			

N, Number of participants in each group with a given characteristic; SD, standard deviation; df, degrees of freedom. †, for continuous variables, summary statistics are expressed by means and SDs; for categorical variables, summary statistics are expressed by number of participants with a given characteristic and percentages. ‡, between-group comparisons of continuous variables are presented as mean differences (Δ), 95% CI, and corresponding t-statistics. Association categorical variables and Luminal A vs. B cancer have been presented as proportions and corresponding χ^2^ statistics.

**Table 2:** Results of univariate and multivariate logistic regression models predicting likelihood of Luminal B breast cancer.

Model	Variable	β	SE	t-Statistic	*p*-Value
1	Intercept	0.361	0.038	9.411	<0.001
	BMI	0.003	0.001	2.595	0.010
2	Intercept	0.590	0.046	12.729	<0.001
	Age	−0.002	0.001	−2.983	0.003
3	Intercept	0.494	0.021	23.690	<0.001
	Menopause	−0.053	0.023	−2.280	0.023
4	Intercept	0.596	0.030	19.957	<0.001
	Asian	−0.102	0.065	−1.583	0.114
	Latin American	−0.156	0.049	−3.198	0.001
	Native American	−0.167	0.190	−0.879	0.380
	Pacific Islander	−0.096	0.205	−0.467	0.641
	Other	−0.134	0.068	−1.959	0.050
	Unknown	−0.142	0.038	−3.766	<0.001
	White	−0.156	0.031	−4.969	<0.001
5	Intercept (All other races)	0.441	0.009	47.053	<0.001
	Black/African American	0.154	0.031	4.936	<0.001
6	Intercept	−0.511	0.181	−2.816	0.005
	BMI	0.015	0.005	0.648	0.008
	Black/African American	0.600	0.139	4.333	<0.001
	Menopause	−0.218	0.099	−2.194	0.028

SE, Standard error; β, regression coefficient.

**Table 3:** Results of mediation analyses examining whether association between Black/African American and Luminal B breast carcinoma is mediated by BMI.

Step	Dependent Variable	Predictor	β	SE	t-Statistic	*p*-Value
***Mediation analysis without a covariate***						
1	Luminal B breast cancer	Intercept	0.441	0.009	47.053	<0.001
		Black/African American	0.154	0.031	4.936	<0.001
2	BMI	Intercept	29.988	0.144	208.187	<0.001
		Black/African American	2.295	0.482	4.764	<0.001
3	Luminal B breast cancer	Intercept	−0.396	0.105	−3.783	<0.001
		Black/African American	0.386	0.857	4.501	<0.001
		BMI	0.008	0.003	2.399	0.016
***Mediation analysis including menopausal state as a covariate***						
1	Luminal B breast cancer	Intercept	−0.064	0.056	−1.140	0.255
		Black/African American	0.400	0.085	4.681	<0.001
		Menopause	−0.109	0.062	−1.780	0.075
2	BMI	Intercept	28.751	0.318	90.522	<0.001
		Black/African American	2.356	0.480	4.905	<0.001
		Menopause	1.522	0.349	4.364	<0.001
3	Luminal B breast cancer	Intercept	−0.314	0.113	−2.787	0.005
		Black/African American	0.380	0.086	4.429	<0.001
		BMI	0.009	0.003	2.558	0.011
		Menopause	−0.123	0.062	−1.990	0.047

SE, Standard error; β, regression coefficient.

## Data Availability

The data presented in this study are available on request from the corresponding authors.
